# Three-dimensional composite substrate based on pyramidal pitted silicon array adhered Au@Ag nanospheres for high-performance surface-enhanced Raman scattering

**DOI:** 10.1515/nanoph-2024-0354

**Published:** 2024-09-27

**Authors:** Wei Zhang, Siqi Liu, Sijia Jiang, Jiahang Zhang, Hongtao Ma, Liang Xu, Mingyu Yang, Ding Ma, Qingbin Jiao, Xin Tan

**Affiliations:** Fine Mechanics and Physics, Chinese Academy of Sciences, Changchun Institute of Optics, Changchun 130033, China; University of the Chinese Academy of Sciences, Beijing 100049, China

**Keywords:** 3D composite SERS substrate, pyramidal pitted silicon, Au@Ag nanospheres, pathogen detection

## Abstract

As a noninvasive and label-free optical technique, Raman spectroscopy offers significant advantages in studying the structure and properties of biomacromolecules, as well as real-time changes in cellular molecular structure. However, its practical applications are hindered by weak scattering responses, low signal intensity, and poor spectral uniformity, which affect the subsequent accuracy of spectral analysis. To address these issues, we report a novel surface-enhanced Raman scattering (SERS) substrate based on a pyramidal pitted silicon (PPSi) array structure adhered with Au-shell Ag-core nanospheres (Au@Ag NSs). By preparing a highly uniform PPSi array substrate with controllable size and arrangement, and constructing SERS-active Au@Ag NSs on this substrate, a three-dimensional (3D) composite SERS substrate is realized. The enhancement performance and spectral uniformity of 3D composite SERS substrate were examined using crystal violet (CV) and Rhodamine 6G (R6G) molecules, achieving a minimum detectable concentration of R6G at 10^−9^ M and the analytical enhancement factor (AEF) of 4.2 × 10^8^. Moreover, SERS detection of biological samples with varying concentrations of *Staphylococcus aureus* demonstrated excellent biocompatibility of the SERS substrate and enabled quantitative analysis of bacterial concentration (*R* ^2^ = 99.7 %). Theoretical simulations using finite-difference time-domain (FDTD) analysis were conducted to examine the electromagnetic field distribution of the three-dimensional SERS composite substrate, confirming its local electric field enhancement effect. These experimental and theoretical results indicate that the Au@Ag NSs/PPSi substrate with a regulable pyramidal pitted array is a promising candidate for sensitive, label-free SERS detection in medical and biotechnological applications.

## 1 Introduction

The environment and food harbor a diverse range of pathogenic bacteria, characterized by rapid mutation rates and high infectivity, posing severe threats to human life and health [1]. Early, rapid, and accurate detection of these pathogens is crucial for effective monitoring, prevention, and control of epidemics, thereby safeguarding human health. However, conventional methods for pathogen detection such as isolation-culture identification technology, immune diagnosis technology, and molecular biology diagnosis technology suffer from drawbacks including high costs and time-consuming procedures [2], rendering them inadequate for on-site pathogen detection [3]. Therefore, there is an urgent need to develop highly sensitive label-free field detection methods that are both versatile [4], which would significantly impact clinical diagnostics as well as biosafety measures in public health.

Surface-enhanced Raman scattering (SERS) technology has been widely used in rapid substance analysis and detection because of its inherent advantages [5], [6], [7], including simplified sample collection, expedited sampling process, nondestructive nature of samples, and elimination of complex pretreatment steps. Especially in the field of pathogenic bacteria detection, SERS technology has shown great potential. This technique enables characterization of bacterial molecular structures at the single-molecule level [8] and facilitates label-free detection with high speed [9], [10], [11], [12]. Integration with commercially available portable Raman spectrometers allows for facile on-site real-time pathogen detection [13], thereby offering extensive research opportunities and promising applications in clinical diagnosis, biosafety measures, public health management, and other related domains.

In terms of the enhancement principle of SERS technology, it is widely acknowledged as the fusion effect of electromagnetic mechanism (EM) [14] and chemical mechanism (CM) [15]. EM excited by surface plasmon resonance in tiny metals plays a dominant role [16], leading to an overall enhancement ranging from 10^6^ to 10^10^. However, for CM, only a modest 10–100× enhancement can be achieved, which is an enhancement effect similar to resonance Raman scattering produced by electron coupling of some molecules adsorbed on the surface. During SERS analysis and detection, the molecular specific Raman signal was mainly enhanced due to plasmon resonance on nearby metal surface [17]. The Raman signals obtained from SERS substrates are mainly derived from “hot spots” generated by gaps, sharp edges, and nanostructure tips with diverse shapes and sizes [18], [19], which significantly enhance local electromagnetic fields and enable highly sensitive molecule analysis within these “hot spots” [20], [21]. By continuously optimizing and preparing special metal structures to construct one-dimensional or two-dimensional substrates, systems have been shown to result in a large increase in the number of “hot spots” that enhance SERS activity, which include spherical particles, rod-like particles, cubic particles, octahedral particles, triangular thin plates, and double-cone Au and Ag nanoparticles [22], [23], [24]. Among them, spherical nanoparticles are considered classical isotropic particles that offer advantages in generating uniform signals. Compared to polyhedral nanocrystals, the electrokinetic response of spherical nanoparticles can be analytically solved using Mie theory within the classical electromagnetic framework. Moreover, the use of spherical particles is much more convenient for basic theoretical studies [25]. Moreover, the integration of multicomponent materials in core–shell nanospheres can synergistically enhance their properties [26], [27]. By utilizing highly monodisperse and stable Au nanocrystals as the core, dimensional homogeneity of the product is achieved. Additionally, incorporating Ag as the shell enhances SERS activity due to its superior performance [26].

However, the one-dimensional SERS substrate based on Au, Ag, and other noble metal nanoparticle sol is typically confined to a specific or single point location. This limitation in stability and controllability has restricted its application range [28]. Two-dimensional layered materials are being considered as promising SERS substrates due to their cost-effectiveness and nontoxic properties. However, compared with traditional precious metal substrates, they exhibit lower enhancement factors and detection limits [29]. Despite the many advantages of these low-dimensional substrates in SERS applications, such as lower cost and simple preparation, their sensitivity is often compromised by poor structural controllability and limited electromagnetic hot spot density.

Compared to low-dimensional substrates, 3D SERS substrates offer a larger surface area, which is more conducive to enhancing molecular enrichment near metal nanoparticles and increasing the number of hot spots per unit projected area [30], [31]. This effectively amplifies the light field near metal nanoparticles, providing significant advantages in SERS applications [32]. As a result, various 3D SERS substrates with rigid structures based on silicon and quartz have emerged in recent year. Zhang et al. utilized a simple and cost-effective method to obtain pyramidal Si (PSi) SERS substrate with a nanoporous pyramid array structure, achieving excellent enhancement effects for the 3D SERS substrate based on PSi [33]. However, the size and distribution of PSi obtained by direct corrosion of silicon with alkali solution were formed randomly, leading to uncontrollable period and unguaranteed uniformity of arrangement. Consequently, the scattering response uniformity of the substrate cannot be controlled. Therefore, it is necessary to develop an approach for manufacturing well-ordered pyramids arrays with highly uniformed shapes over a large area. In our work, we propose a relatively simple method to fabricate regularly arranged and period-controlled PPSi array, which allows for adjustment of the structure size and distribution of the array to ensure substrate uniformity. Additionally, compared with slopes in the open area of PSi, valleys enclosed by PPSi slopes can generate effective oscillation of incident laser light as an amplifier and introduce larger electric field intensity for plasmon resonance.

Herein, a sensitive, homogeneous, and biocompatible 3D composite SERS substrate based on PPSi array was presented, which could achieve rapid SERS detection of pathogenic bacteria. The PPSi array structure, serving as a rigid substrate, was achieved through the wet etching method using the single crystal property of silicon. Au@Ag nanospheres were self-assembled on the surface of PPSi to create a large number of hot spots in three-dimensional space, effectively enhancing the Raman signal of the target object. The enhanced properties and homogeneity of the prepared SERS substrates were confirmed using the reporter molecule R6G. Additionally, different concentrations of *Staphylococcus aureus* were successfully detected using the SERS substrate, demonstrating its efficacy in practical biological sample measurements.

## 2 Experimental section

**Figure 1:**
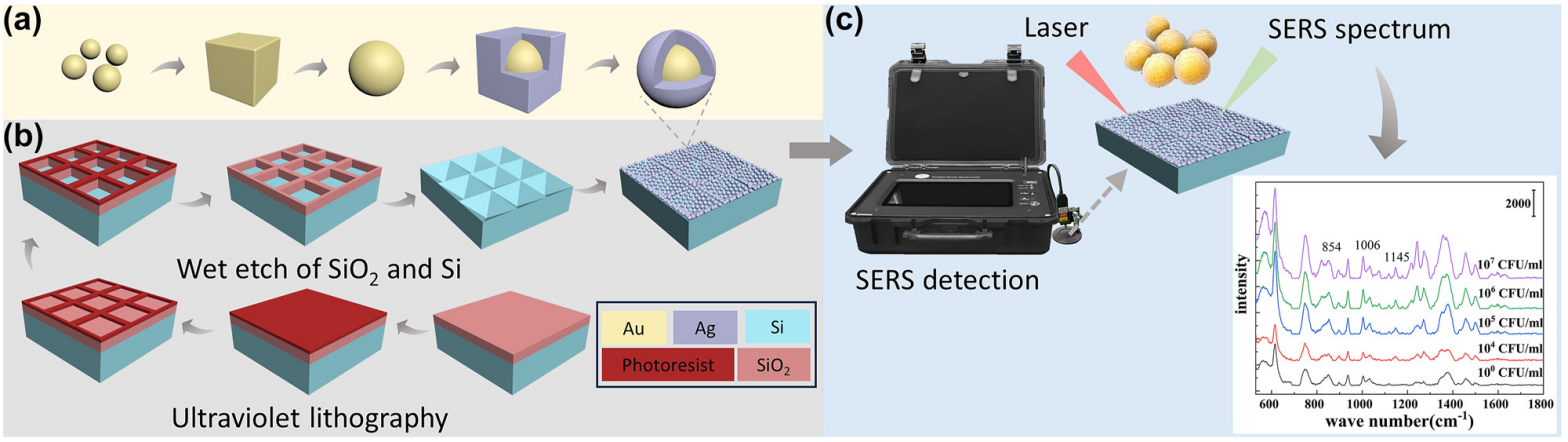
The preparation of the Au@Ag NSs/PPSi SERS substrate and the SERS detection process. (a) Schematic diagram showing the structure and preparation of the Au-cored Ag-shell nanospheres. (b) Schematic diagram showing the preparation of the pyramidal pitted silicon substrates. (c) Schematic diagram showing the process of the SERS detection with Au@Ag NSs/PPSi SERS substrate.

In this work, Au@Ag NSs were synthesized through seed growth and oxidation etching (Figure 1(a)), leading to enhanced strengthening properties attributed to the synergistic effects of Au and Ag. Subsequently, we utilized photolithography and wet etching techniques to create a regularly patterned, period-controlled PPSi array substrate in order to provide an enlarged adhesion area and a more robust solid support (Figure 1(b)). A dense and highly uniform monolayer metal film was produced using the interfacial self-assembly method, which was then transferred onto the PPSi substrate to fabricate a 3D composite SERS substrate based on PPSi attached Au@Ag NSs. The resulting SERS substrate exhibited exceptional uniformity, tunable morphology, and high sensitivity. When coupled with a portable Raman spectrometer, this composite SERS substrate enabled the detection of reporter molecules and *S. aureus* (Figure 1(c)).

### 2.1 Pyramidal pitted silicon preparation

First, the <100> polished silicon coated with 300 nm SiO_2_ was cleaned on the surface, dried with nitrogen, and baked in the oven at 90 °C to remove the moisture in the silicon. The appropriate amount of positive photoresist drops is added to the center area of the silicon wafer. Then, a three-stage spin coating process was applied successively for 5 s at 500 rmp, 30 s at 4,000 rmp, and another 5 s at 500 rmp. The silicon wafer coated with the photoresist was then baked again at a constant temperature to ensure that all solvent in the photoresist fully volatilized.

After natural cooling to room temperature, ultraviolet lithography exposure machine with mask plate exposure was performed. Subsequently, development took place in a solution containing 3 ‰ sodium hydroxide followed by thorough cleaning with deionized water to complete fixing. The silicon wafer was then baked at a constant temperature for 20–30 min. Afterward, BOE solution (buffer oxide etching solution, 40 % NH_4_F aqueous solution to 49 % HF volume ratio 7:1) was used to etch the exposed SiO_2_ on the silicon wafer. Once etching was complete, acetone was used to remove any remaining photoresist from the surface before immersing it into heated KOH solution (12.5 %) in a water bath set between temperatures of 55–60 °C for anisotropic wet etching until a pyramidal pit structure had formed on the silicon wafer (reaction time of approximately 40 min for every 10 μm). Finally, the silicon wafer underwent immersion in BOE solution once more to remove any remaining SiO_2_ mask from its surface, resulting in the production of PPSi substrates.

### 2.2 Synthesis process of Au@Ag NSs

The seed growth method is applicable for the preparation of various types of nanomaterials, allowing for precise control over the size and morphology of nanoparticles [34]. The fabrication of Au-core Ag-shell nanostructures involves a stepwise process of seed growth and oxidative etching to achieve the desired core–shell structure and shape tuning.

Initially, 0.6 mL of strong reducing agent NaBH_4_ (10 mM) was used in a *CTAB* system (10 mL, 100 mM) to reduce the precursor HAuCl_4_ (0.25 mM), resulting in the aggregation of 0-valent Au atoms into nanoclusters (3 nm). Subsequently, 1.5 mL of weak reducing agent *AA* (100 mM) was employed to further stabilize the reduction of chloroauric acid (2 mL, 0.5 mM) on the clusters, leading to the growth of 10 nm Au seeds. The solution containing these 10 nm Au seeds was then collected through centrifugation and dispersion [35].

Then, in the CTAC (20 mL, 100 mM) system, the reducing agent *AA* (1.3 mL, 10 mM) continued to slowly reduce HAuCl_4_ (20 mL, 0.5 mM) on the Au seed surface, resulting in uniform growth of Au. Surfactant CTAC can be used as a soft template to guide Au along specific crystal faces, ultimately forming a regular cubic morphology [36]. To reduce the introduction of other ions into the system, HAuCl_4_ (250 μL, 10 mM) was used as the oxidant to preferentially etch the sharp edges and corners of the nanocubic, converting the Au nano cubes into 80 nm diameter highly spherical nanoballs.

In the final step, the reducing agent *AA* (2.5 mL, 100 mM) was utilized to gradually reduce the precursor AgNO_3_ (0.5 mM, 50 mL), resulting in the formation of Ag atoms that deposited onto the uniform nano Au nucleus. Similarly, within the CTAC (50 mL, 20 mM) system, chloride ions effectively shielded the <100> facet, allowing for Ag growth along the Si <111> facet and leading to nanostructures with a regular cubic shape. By employing hydrogen chloride at a concentration of 0.2 % as an etching agent, the Au@Ag nano cubes underwent transformation into Au@Ag NSs. The degree of etching on the Ag shell depended solely on the concentration of the etching agent and not on time duration; this method provides effective control over the etching process [37].

### 2.3 Self-assembly of Au@Ag NSs monolayer film

The prepared Au@Ag NSs solution was centrifuged and then dispersed into a 1 % mass ratio PVP ethanol solution to convert CTAC-coated Au@Ag NSs into PVP-coated ones. After another round of centrifugation and dispersion in ethanol, the nanoparticles were placed in a CH_2_Cl_2_ oil system in a centrifuge tube, with an appropriate amount of deionized water added. Following shaking and oscillating, a loosely packed metal film formed at the interface. Subsequently, hexane was added to create an oil–water–oil three-phase system. By tilting and rotating the centrifuge tube on a small scale, the nanoparticles spontaneously move to the hexane/water interface due to interfacial tension gradients, resulting in a dense monolayer film with bright metallic luster. This film was then transferred onto PPSi substrates for assembly into composite SERS substrates.

### 2.4 Substrate characterization

The surface morphology and size distribution of the pyramidal pitted silicon substrate and the Au@Ag nanoparticle structure were characterized using a scanning electron microscope (SEM, Zeiss AURIGA) at an accelerating voltage of 5 kV. The composition and elemental content of the substrate microregion were analyzed using an X-ray energy dispersive spectrometer (EDS, Oxford X-Max) at an accelerating voltage of 20 kV. The prepared nanoparticles were respectively drop-cast on a TEM grid to perform high-resolution morphology characterization and EDX element mapping using a transmission electron microscope (TEM, FEI Talos F200X G2) operating at 200 kV. Raman mapping was carried out by a confocal microprobe Raman system (Horiba Odyssey, 785 nm). The Raman scattering spectrum was measured using a portable Raman spectrometer (Optosky ATR3000, excitation wavelength: 785 nm, adjustable excitation power: 0–500 mW).

### 2.5 Bacterial sample culture and SERS detection

The preserved strain was inoculated onto Luria-Bertani (LB) slope culture medium for preculture to obtain the first-generation strain for further experiments. Subsequently, the cultured bacteria were inoculated into LB liquid culture medium and shaken at 220 rpm on a shaker at 37 °C for 24 h. Following this, the cultured liquid was centrifuged to remove the supernatant, and 5 mL of physiological saline was added for a second centrifugation to wash the bacteria. Afterward, 2 mL of bacterial suspension was prepared using physiological saline, and 300 μL was added to a spectrophotometer cuvette and diluted tenfold. The OD value of the bacterial suspension at 600 nm was measured to be 0.287 using a spectrophotometer, indicating that the initial undiluted concentration of *S. aureus* was 2.87 × 10^9^ CFU/mL. The bacterial suspension at concentrations ranging from 10^4^ to 10^8^ CFU/mL was then stepwise diluted. Following this, drops containing 20 μL of the 10^4^ − 10^8^ CFU/mL *S. aureus* suspension were placed on to the prepared composite SERS substrate and allowed to dry naturally at room temperature. SERS spectra were collected using a portable Raman spectrometer (wavelength 785 nm, intensity 100 mW, integration time 2000 ms) after the samples were dried. Five points were randomly selected from each sample for detection to obtain the average value as the final result. At the same time, the blank sample prepared by adding 20 μL of physiological saline to the composite SERS substrate was collected under the same conditions.

### 2.6 Detection of Raman reporters by SERS substrate

The SERS substrates were evaluated for enhancement and uniformity using the Raman reporter molecule R6G. A 10^−1^ M R6G solution was prepared and stepwise diluted to obtain a low concentration solution of 10^−4^ − 10^−9^ M. The bare silicon wafer was treated with a 20 μL 10^−1^ M R6G solution, dried at room temperature, and then analyzed using a portable Raman spectrometer with an excitation wavelength of 785 nm, excitation intensity of 100 mW, and integration time of 5,000 ms as the control group. Similarly, a 20 μL solution of 10^−4^ − 10^−9^ M R6G was applied to the prepared SERS substrate, dried naturally at room temperature, and then analyzed using a portable Raman spectrometer under the same conditions. To ensure reliable results, spectra were collected from five random points each time and averaged for the final result.

## 3 Results and discussion

### 3.1 Optimization and characterization of PPSi substrates

**Figure 2:**
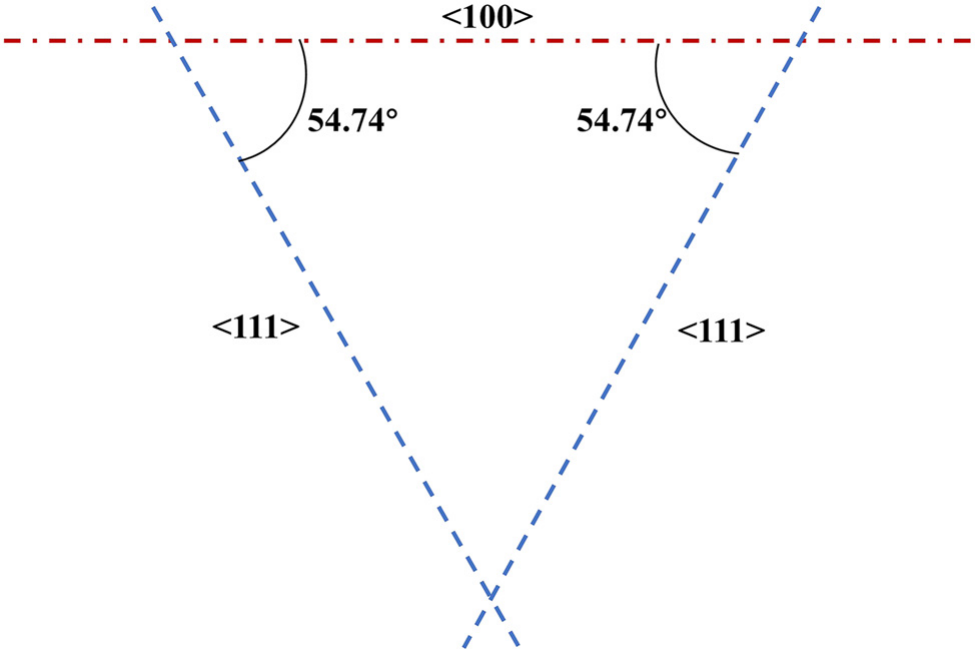
Schematic diagram showing the angle of <100> silicon wafer direction.

The crystal structure of silicon is a cubic crystal system, where the most common crystal directions are <100> and <111>. Due to the difference of crystal structure, the reaction rate of <100> and <111> crystalline orientation silicon to hydroxide ion is different, which leads to the anisotropic etching phenomena. Generally, the <100> crystal direction reacts more readily with hydroxide ions compared to the <111> crystal direction, resulting in a faster etching rate. As shown in Figure 2, the angle between the <111> plane and the <100> plane of the <100> silicon wafer is 54.74° [38]. When a mask with regularly arranged square holes is applied on the surface of silicon using ultraviolet lithography, a PPSi structure with regular arrangement and uniform shape is formed on the silicon wafer through anisotropic etching process.

**Figure 3:**
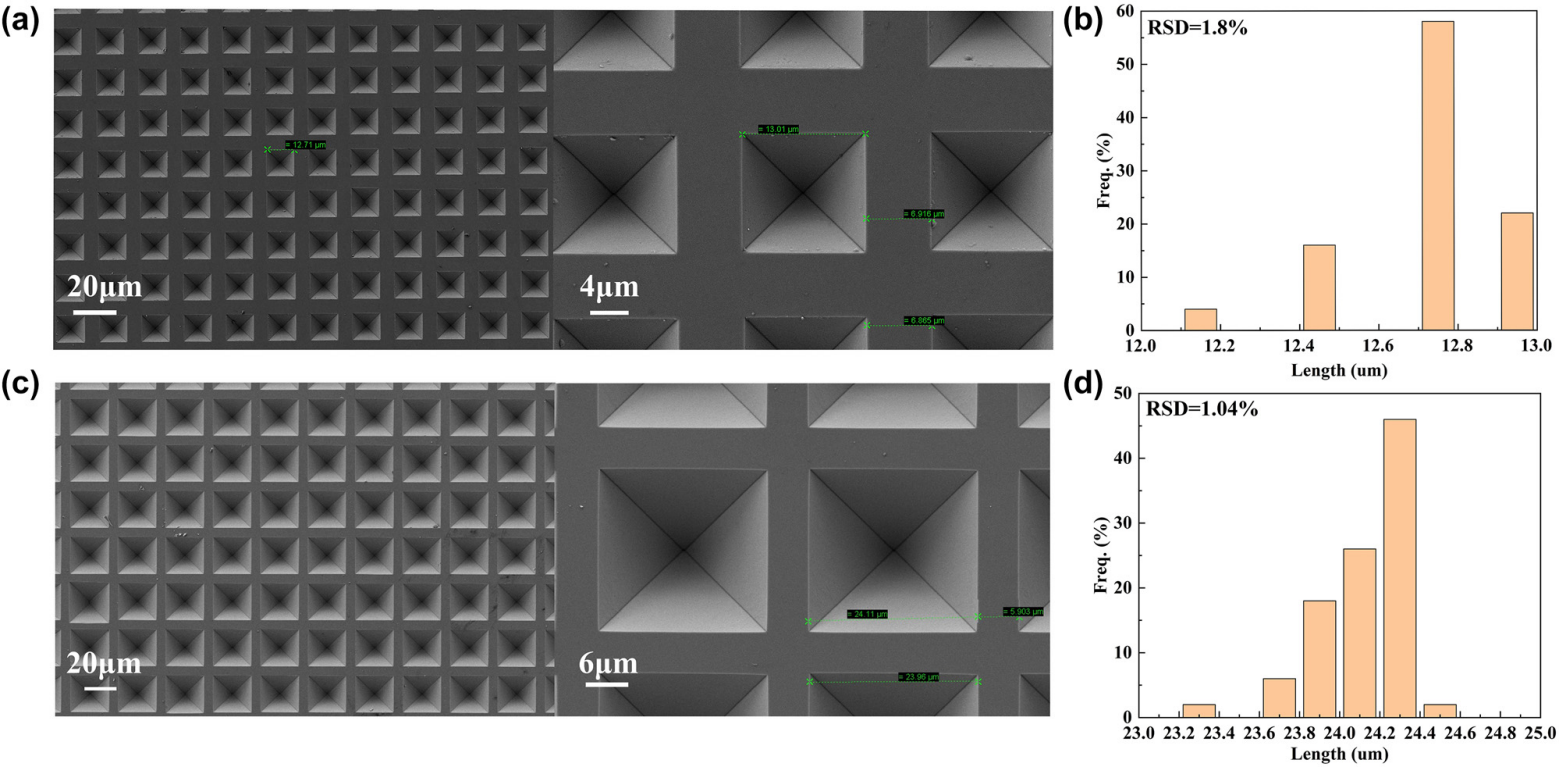
SEM picture of the PPSi substrates. (a) PPSi prepared by a 10 μm square hole with a spacing of 10 μm mask. (b) The size distribution histogram of PPSi prepared by a 10 μm square hole with a spacing of 10 μm mask. (c) PPSi prepared by a 20 μm square hole with a spacing of 10 μm mask. (d) The size distribution histogram of PPSi prepared by a 20 μm square hole with a spacing of 10 μm mask.

Figure 3(a) and (c) show the SEM images of the PPSi structures after etching for different periods. It can be observed that the silicon wafer is etched along the <111> crystal orientation, and the four <111> crystal facets extend downward and converge to form regular pyramid-shaped pits. The PPSi structure array was evenly distributed, with intact pit surfaces and complete structures, where the edges, vertices, and bottom edges were clearly defined, indicating a satisfactory etching effect. The Nano Measure 1.2 software was used to measure the bottom side lengths of 50 randomly prepared PPSi structures and calculate the size distribution. As depicted in Figure 3(b) and (d), the size distribution of the PPSi array is remarkably uniform, with a very small relative standard deviation (RSD) of the side lengths (all within 2 %), which indicates that the uniformity of the prepared PPSi array is excellent.

However, there was a reduction in boundary width after etching compared to the mask size, leading to a corresponding increase in the length of the pyramid base while maintaining an unchanged overall period. For instance, a square mask with a side length of 10 μm results in a 13 μm base for the pyramid and reduces the boundary from 10 μm to 7 μm. Similarly, a PPSi etched from a square mask with a side length of 20 μm had a base edge of 24 μm and reduced the boundary from 10 μm to 6 μm. This phenomenon can be attributed to potassium hydroxide solution corroding silica simultaneously as it etches silicon but at a slower rate.

**Figure 4:**
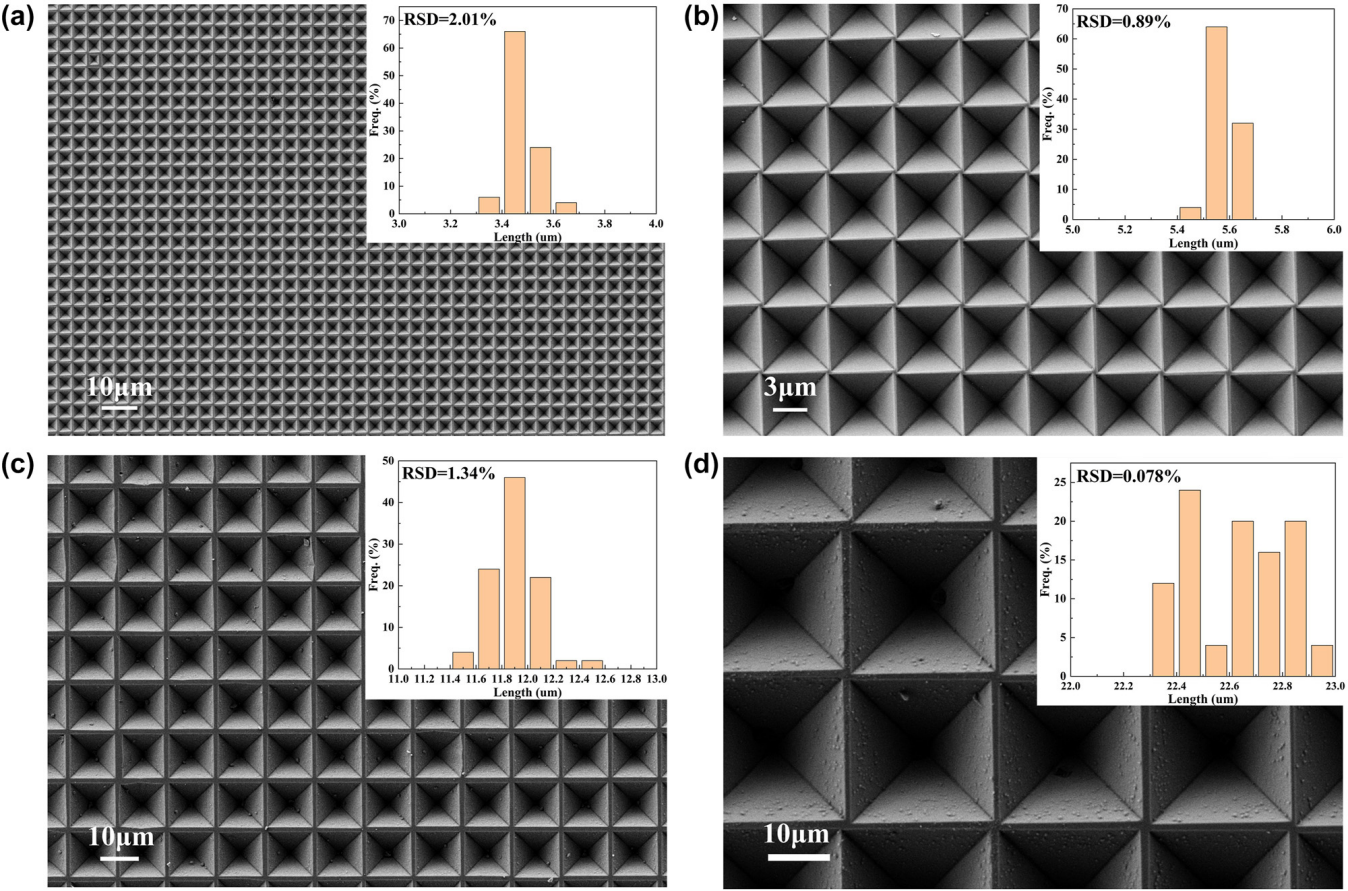
SEM picture of the PPSi substrates. (a) PPSi prepared by a 2 μm square hole with a spacing of 2 μm mask. (b) PPSi prepared by a 4 μm square hole with a spacing of 2 μm mask. (c) PPSi prepared by a 10 μm square hole with a spacing of 3 μm mask. (d) PPSi prepared by a 20 μm square hole with a spacing of 4 μm mask (the illustrations show the histogram of the size distribution corresponding to the PPSi of different sizes).

If the boundary mask is reduced so that the etching amount of the silica mask reaches a critical value, the width of the boundary platform will be significantly reduced, and the slope area of the PPSi array will be further increased as it becomes approximately boundary free. The size of the mask plate was optimized based on actual experimental data. The spacing between 2 μm and 4 μm square holes was adjusted to 2 μm (due to limitations in the mask plate preparation process), while the spacing between 10 μm square holes was set to 3 μm, and the spacing between 20 μm square holes was set to 4 μm. Figure 4 depicts the SEM image of a base prepared using this optimized mask plate, showing that the base length (boundary width) of pyramids measures at 3.5 μm (0.5 μm), 5.6 μm (0.4 μm), 11.9 μm (1.1 μm), and 22.6 μm (1.4 μm). Additionally, corresponding pyramids depths were measured at 2.46 μm, 4 μm, 8.4 μm, and 16 μm, respectively. At this point, the boundary width has been significantly reduced, yet the etching effect on the boundaries remains satisfactory, with sharp and clear edges without any collapse. The size and morphology of the PPSi array exhibit good uniformity.

### 3.2 PPSi substrates with deposition of Au@Ag nanoparticles characterization

Au@Ag NSs were assembled into a highly homogeneous monolayer film using the liquid–liquid interface self-assembly technique. This film was then constructed on a PPSi substrate, utilizing the Marangoni effect. The result was a 3D SERS substrate (Au@Ag NSs/PPSi) with tunable shape and position.

Before interfacial self-assembly, the prepared nanoparticles need to undergo ligand exchange. Due to the amphoteric nature of CTAC and its interaction with both aqueous and organic phases, it is challenging for CTAC-coated Au@Ag NSs to complete self-assembly directly at the interface. Therefore, PVP-coated Au@Ag NSs need to be prepared through a ligand exchange process.

**Figure 5:**
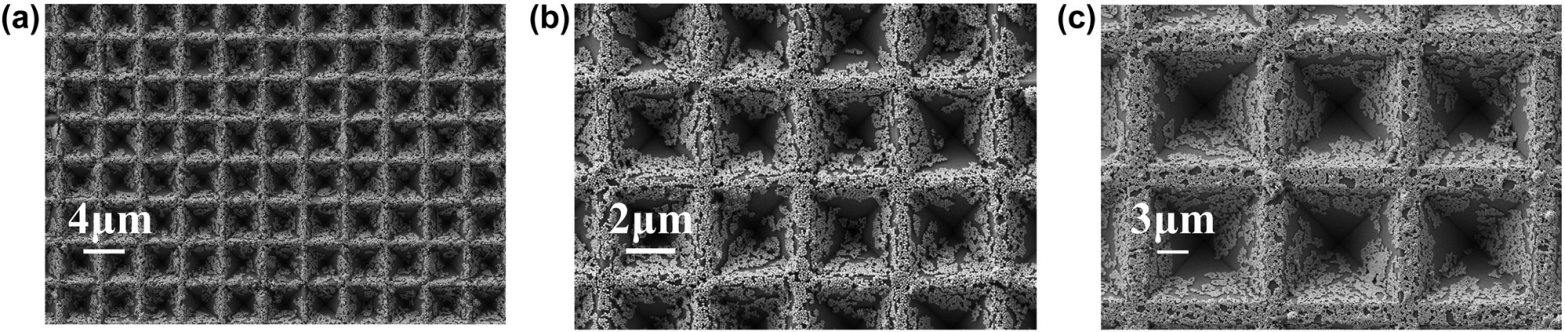
SEM picture of the PPSi substrates with deposition of Au@Ag nanoparticles. (a) PPSi prepared by a 2 μm square hole with a spacing of 2 μm mask. (b) PPSi prepared by a 4 μm square hole with a spacing of 2 μm mask. (c) PPSi prepared by a 10 μm square hole with a spacing of 3 μm mask.

In a water–oil system where the oil phase density is higher than that of the water phase, dichloromethane and water were found to be immiscible, causing the nanoparticles to remain in the organic oil phase. Consequently, they were adsorbed at the interface of the two phases, forming a random, nontightly packed single-layer metal film. However, upon adding another oil phase such as *n*-hexane with a lower density than water, driven by the interfacial tension gradient of the oil–water–oil three-phase system, the nanoparticles can spontaneously climb from the lower interface to the upper interface. This allows for their transfer and compression, resulting in a single layer of metal nanoparticles with close packing state distribution and bright metallic luster. The membrane assembled by Au@Ag NSs was transferred and constructed on PPSi to obtain 3D SERS substrate. The SEM images in Figure 5 showed composite substrates assembled with PPSi substrates of different sizes. It can be observed from these images that Au@Ag NSs are effectively attached to the PPSi substrate with regular morphology and roughly uniform distribution. Overall, this indicates that composite substrate morphology was excellent for further study purposes.

**Figure 6:**
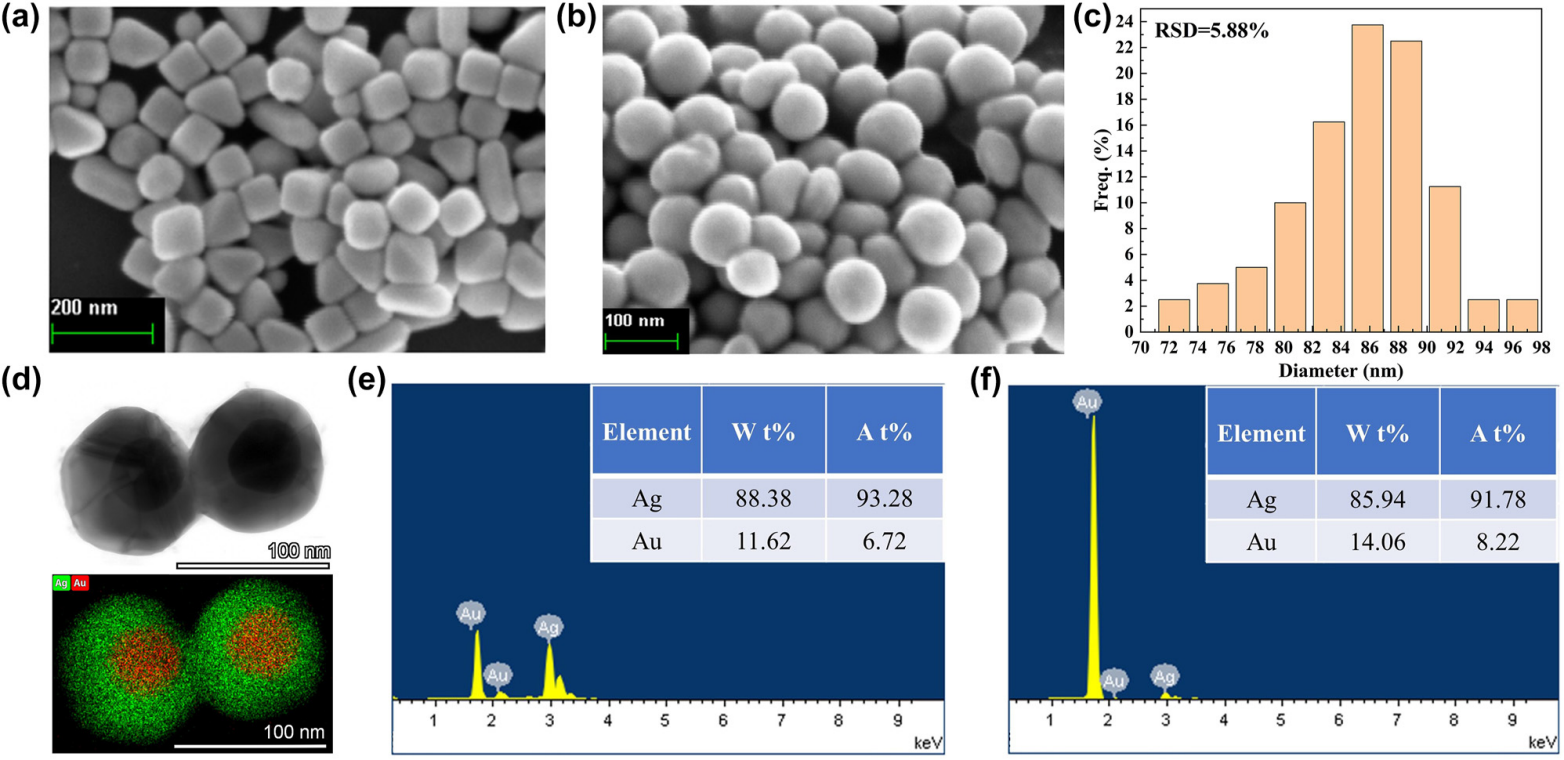
Characterization of Au@Ag nanoparticles. (a) The SEM image of Au@Ag nano cubes. (b) The SEM image of Au@Ag NSs. (c) The size distribution histogram of Au@Ag NSs. (d) The TEM image and elemental mapping of Au@Ag NSs. (e) The EDS spectral line of Au@Ag nano cubes. (f) The EDS spectral line of Au@Ag NSs.

In Figure 6(a) and (b), the prepared Au@Ag nanocubes and Au@Ag NSs were depicted. The Nano Measurer 1.2 software was utilized to measure both random diameter and size distribution for 100 prepared nanospheres as shown in Figure 6(c). Statistical calculation determined that these nanospheres had an average diameter of 85 nm when assembled on a composite substrate with an RSD of 5.88 %. This uniform size distribution indicated consistency among prepared nanospheres. To verify the Au@Ag core–shell structure, high-resolution morphology characterization and elemental mapping using a FEI Talos F200X G2 TEM system operating at 200 kV were performed. The core–shell structure of nanoparticles can be clearly characterized, and the element mapping visually demonstrates the nanostructure of gold core silver shells in Figure 6(d). Figure 6(e) and (f) further reveal the EDS spectral line to Au@Ag NCs and Au@Ag NSs, a comparison of the ratio of Ag to Au content between nanocubes revealed that after etching there was a decrease in weight ratio due to etching away of sharp edges from the Ag shell.

### 3.3 Enhance performance and uniformity of the SERS substrate

**Figure 7:**
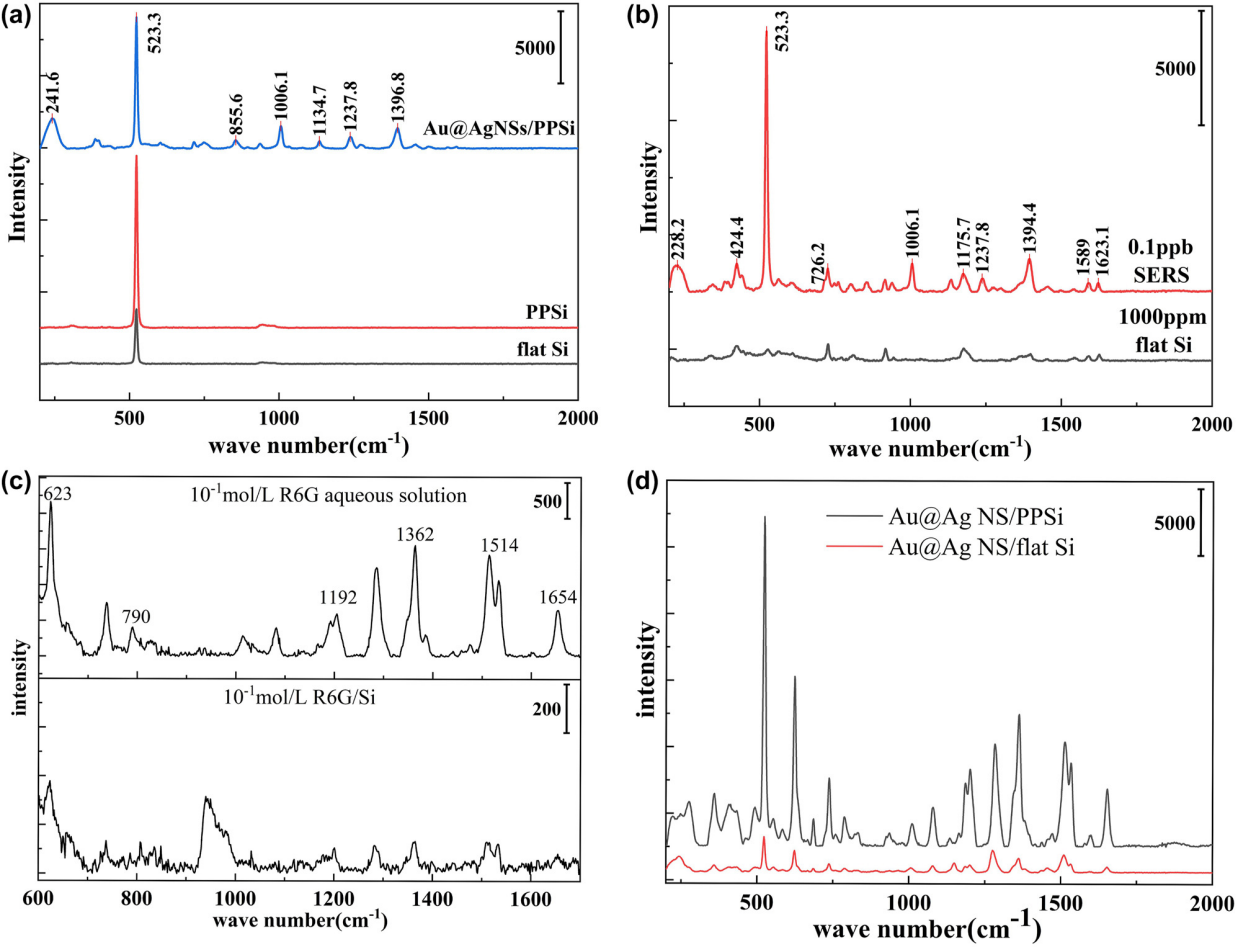
SERS spectra of SERS substrate with different probe molecules. (a) Raman spectra of Si, PPSi, and Au@Ag NSs/PPSi. (b) Raman spectra of 1,000 ppm CV and SERS spectra of 0.1 ppb CV. (c) Raman spectra of 10^−1^ M R6G solution. (d) SERS spectra of 10^−4^ M R6G on Au@Ag NSs/flat Si and Au@Ag NSs/PPSi.

In Figure 7(a), the distinct Raman responses of Si wafer, PPSi, and Au@Ag NSs/PPSi were clearly observed. The peak at around 523 cm^−1^ is attributed to the silicon lattice vibration. In comparison to the untreated Si wafer, the Raman spectrum of PPSi exhibits a significant enhancement in the intensity of the 523 cm^−1^ peak, which was twice as strong as that of the silicon wafer. For the 3D composite SERS substrate, characteristic peaks at 241, 1,006, 1,238, and 1,397 cm^−1^, attributed to the metal nanoparticles (Au@Ag NSs), were detected in the spectrum. Simultaneously, due to reduced exposed silicon area after attachment of Au@Ag NSs, there is a decrease in intensity of the 523 cm^−1^ peak. We applied CV solutions at concentrations of 1,000 ppm onto silicon wafer and 0.1 ppb onto a 3D composite SERS substrate, respectively. The major characteristic peaks of CV molecules at 1,589 cm^−1^ and 1,623 cm^−1^, which correspond to the in-plane stretching vibrations of the C–C bond, are used as subjects of study typically. As shown in Figure 7(b), the characteristic peaks of 0.1 ppb CV on the 3D composite SERS substrate can be easily captured, whereas the peak intensity of 1,000 ppm on the silicon wafer remained particularly weak.

Subsequently, we selected Rhodamine 6G (R6G) as a probe molecule to thoroughly investigate the enhancement performance of the Au@Ag NSs/PPSi composite substrate. And 1 mL 10^−1 ^M Rhodamine 6G (R6G) solution was used as the standard, and the characteristic Raman peak of R6G in the region of 300–2000 cm^−1^ was observed. In Figure 7(c), the peak at 623 cm^−1^ corresponds to the C–C–C in-plane vibration of the R6G molecule, while the peaks at 790 and 1,190 cm^−1^ can be attributed to the out-of-plane vibration and in-plane vibration of the C–H bond, respectively. Additionally, peaks observed at 1362, 1514, and 1,654 cm^−1^ can be classified as aromatic C–C tensile vibration modes [39]. As shown in Figure 7(d), the SERS signal of Au@Ag NSs/PPSi was more than ten times that of Au@Ag NSs/flat Si. The comparison confirmed the signal amplification capability of PPSi array structure and its effective effect on SERS performance.

**Figure 8:**
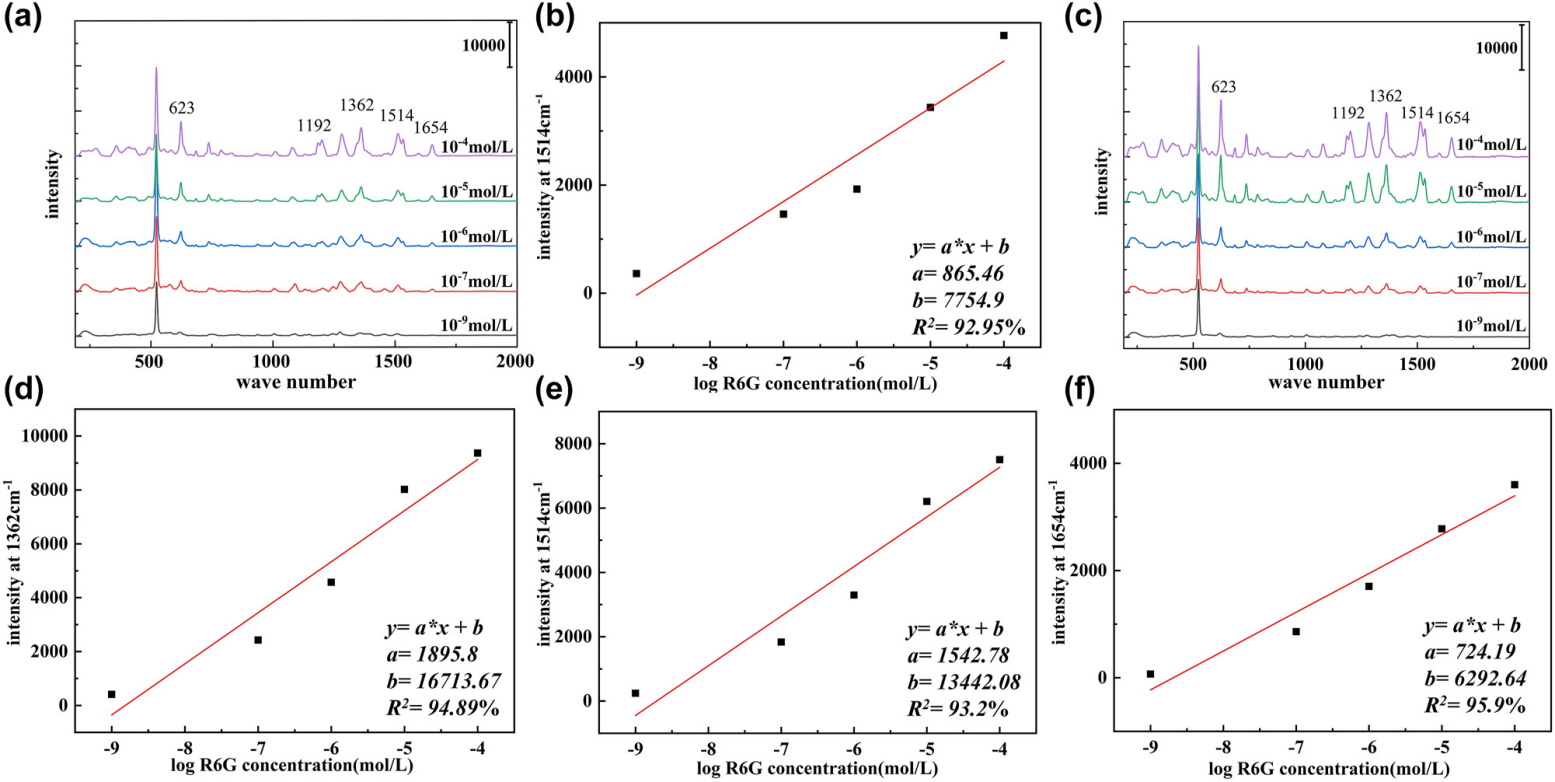
SERS spectra of R6G and  calibration curves  of Raman intensity versus R6G concentration. (a) SERS spectra with R6G concentration of 10^−4^ − 10^−9^ M on the 4 μm composite SERS substrate. (b) The calibration curves of Raman intensity versus R6G concentration (10^−4^ − 10^−9^ M) at 1,514 cm^−1^ on the 4 μm composite SERS substrate. (c) SERS spectra with R6G concentration of 10^−4^ − 10^−9^ M on the 8.4 μm composite SERS substrate. (d) The calibration curves of Raman intensity versus R6G concentration (10^−4^ − 10^−9^ M) at 1,362 cm^−1^ on the 8.4 μm composite SERS substrate. (e) The calibration curves of Raman intensity versus R6G concentration (10^−4^ − 10^−9^ M) at 1,514 cm^−1^ on the 8.4 μm composite SERS substrate. (f) The calibration curves of Raman intensity versus R6G concentration (10^−4^ − 10^−9^ M) at 1,654 cm^−1^ on the 8.4 μm composite SERS substrate.

As shown in Figure 8(a) and (c), when the concentration of R6G test solution decreased from 10^−4^ M to 10^−9^ M, the SERS intensity of R6G molecules gradually decreased with it. However, even at a concentration of 10^−9^ M, the characteristic peak of R6G could still be detected on the composite SERS substrate with the height of 4 μm (Figure 8(a)) and 8.4 μm (Figure 8(c)). As depicted in Figure 8(b), for the 4 μm 3D composite SERS substrate, a good linear SERS dependence was observed between SERS intensity at 1,514 cm^−1^ and R6G concentration ranging from 10^−4^ M to 10^−9^ M showing a high linear regression fitting (*R* ^2^ = 92.95 %). Similarly, as shown in Figure 8(d)–(f), for the 8.4 μm SERS substrate, the peak intensities at 1,362 cm^−1^, 1,514 cm^−1^, and 1,654 cm^−1^ showed linear regression *R* ^2^ values of 94.89 %, 93.2 %, and 95.9 %, respectively, in relation to the R6G concentration. These results demonstrated that the 3D composite SERS substrates can effectively predict analyte concentrations and enable quantitative analysis of molecular quantities.

In order to quantitatively characterize the enhancement performance of the SERS substrate, the enhancement factor of SERS substrate was calculated using formula as follows:

(1) $$AEF=\frac{I_{\text{SERS}}/C_{\text{SERS}}}{I_{\text{normal}}/C_{\text{normal}}}$$

Here, the *I*_SERS_ represents the intensity of 10^−9^ M R6G obtained from the SERS spectrum at 1,362 cm^−1^, while *I*_normal_ denotes the intensity of the normal Raman spectrum of 10^−1^ M R6G at 1,362 cm^−1^. *C*_SERS_ and *C*_normal_ refer to concentrations in SERS and conventional Raman measurements, respectively. The results indicated that the maximum enhancement factor of the SERS substrate with a height of 8.4 μm was found to be 4.2 × 10^8^.

**Figure 9:**
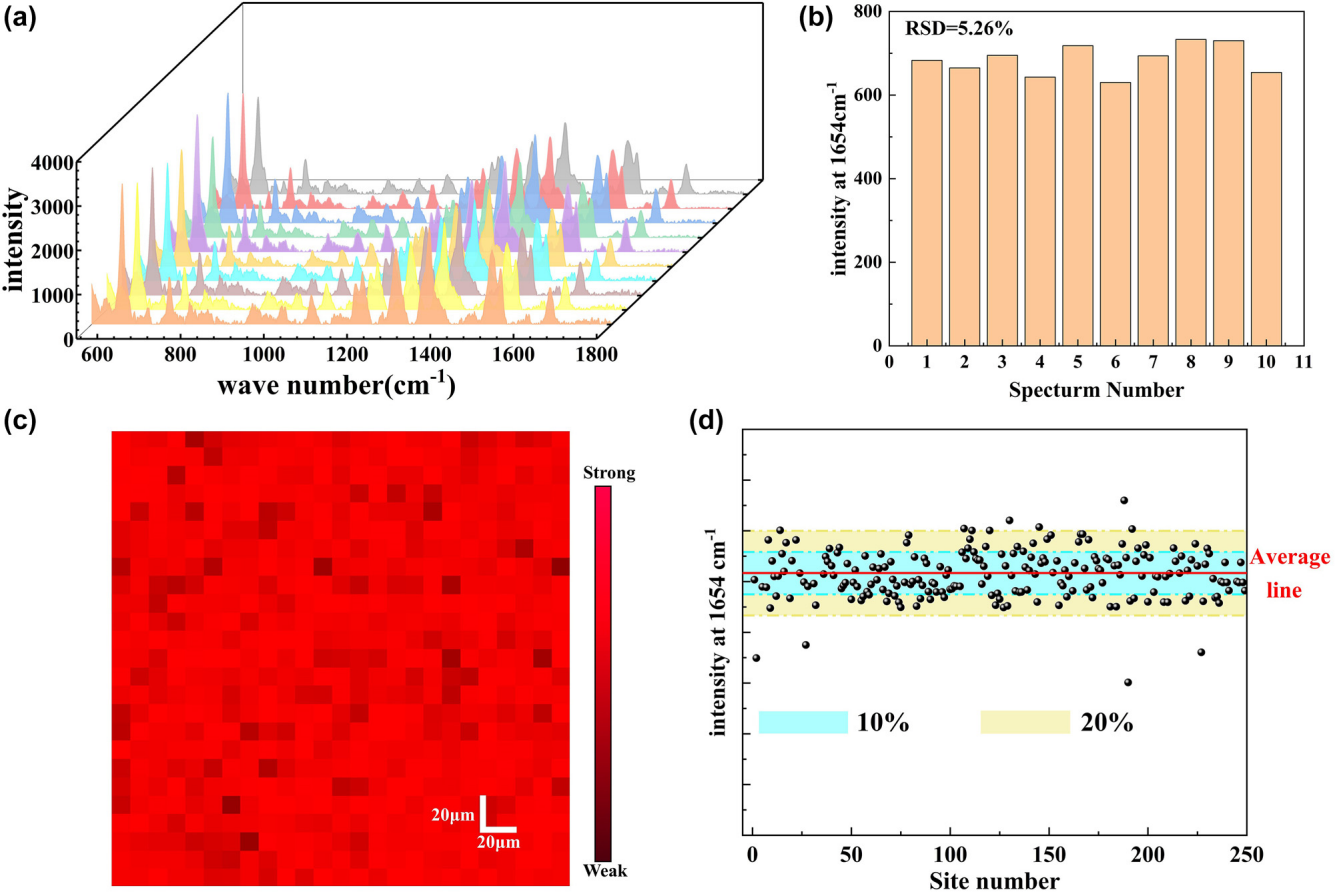
Spectral uniformity characterization of SERS substrate. (a) Randomly select 10 different positions on the SERS substrate to record the SERS signal of 10^−5^ M R6G. (b) The RSD value of the corresponding signal calculated from the characteristic peak of 1,654 cm^−1^. (c) SERS mapping at 1,654 cm^−1^ for 10^−4^ M R6G recorded from the Au@Ag NSs/PPSi substrate. (d) SERS intensity deviation at the 1,654 cm^−1^ calculated with the SERS mapping.

In addition to enhancing the effect, the SERS active substrate also requires good uniformity and reproducibility of SERS signal [40]. To more accurately evaluate the uniformity of Raman signal distribution, a 20 μl 10^−5^ M R6G solution was added to the composite substrate and air-dried at room temperature. SERS spectra of 10 random points were collected under a unified detection environment. The probe focus can move freely on the surface of the substrate, and the spectral trend and peak shape of all points are consistent in Figure 9(a). The uniformity of spectral data reflects the distribution and performance of nanoparticles on the substrate. The RSD of the characteristic peak intensity at the corresponding point 1,654 cm^−1^ is 5.26 % (Figure 9(b)). The Raman mapping taken from a 250 μm × 250 μm area was used to evaluate the signal repeatability. As shown in Figure 9(c), the uniform color of the mapping spectrum at 1,654 cm^−1^ using 10^−4^ M R6G as the probe molecule indicates the excellent signal homogeneity of the Au@Ag NSs/PPSi substrate over a large microscopic area in the detection. Figure 9(d) exhibits the signal intensity deviation of the peak at 1,654 cm^−1^ calculated by using the spectra from the SERS mapping shown in Figure 9(c). More than 70 % of the sites exhibit intensities within the range of *I*_average_ ± *σ*, while over 95 % of the sites have intensities falling within the range of *I*_average_ ± 2*σ*. The RSD of SERS signals is evaluated to be 8.6 %, displaying a good spectral uniformity of the sensor in the microscopic region. The good uniformity of the SERS signal can be attributed to the periodic PPSi array structure and the well-distributed Au@Ag NSs. Homogeneous distribution of nanoparticles on the surface of PPSi enables uniform distribution of hot spots, allowing for effective oscillation by incident laser in creating high-density three-dimensional active hot spots within PPSi troughs. The Au@Ag NSs/PPSi composite structure provided a uniform spatial distribution for analyte molecular detection at designated hot spots.

### 3.4 Application in the detection of pathogenic bacteria

**Figure 10:**
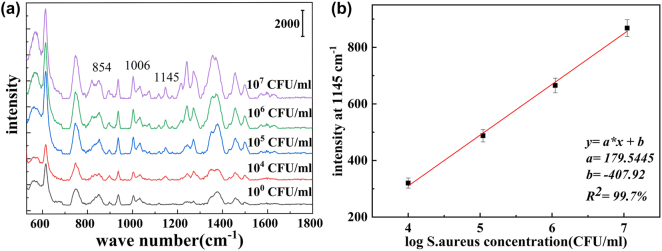
SERS spectra of *S. aureus* and calibration curves of Raman intensity versus *S. aureus*. (a) SERS spectra with *S. aureus* concentration of 10^4^–10^7^ CFU/mL on the composite SERS substrate. (b) The calibration curve of Raman intensity versus *S. aureus* concentration (10^4^–10^7^ CFU/ml) at 1,145 cm^−1^ on the composite SERS substrate.

*S. aureus* samples were utilized to validate the potential for pathogen detection and the efficacy of Raman enhancement. In comparison to the control group, a distinct peak at 1,145 cm^−1^ corresponding to *S. aureus* was identifiable [4]. As the concentration of *S. aureus* increased from 10^4^ CFU/mL to 10^7^ CFU/mL, there was a significant rise in SERS signal intensity with increasing bacterial solution concentration, as depicted in Figure 10(a). Notably, the characteristic peak of *S. aureus* remained detectable even at a concentration of 10^4^ CFU/mL. The SERS dependence on bacterial concentration between 10^4^ CFU/mL and 10^7^ CFU/mL exhibited strong linearity, with a high linear regression fitting *R* ^2^ value of 99.7 % (Figure 10(b)).

### 3.5 FDTD simulation

To better understand the enhancement mechanism and behavior of the Au@Ag NSs/PPSi composite SERS substrate, finite difference time domain (FDTD) simulation was conducted using Ansys Lumerical software to calculate the local enhanced electric fields of Au@Ag NSs/Si and Au@Ag NSs/PPSi. The size of the micro–nano structure in all simulations was determined based on statistical results from experimental measurements. For all array calculations, the background refractive index was set to the refractive index of air. Periodic boundary conditions were applied to the *x* and *y* axes, while PML boundary conditions were used for the *z* axis. The light source utilized was a plane wave with a wavelength of 785 nm.

**Figure 11:**
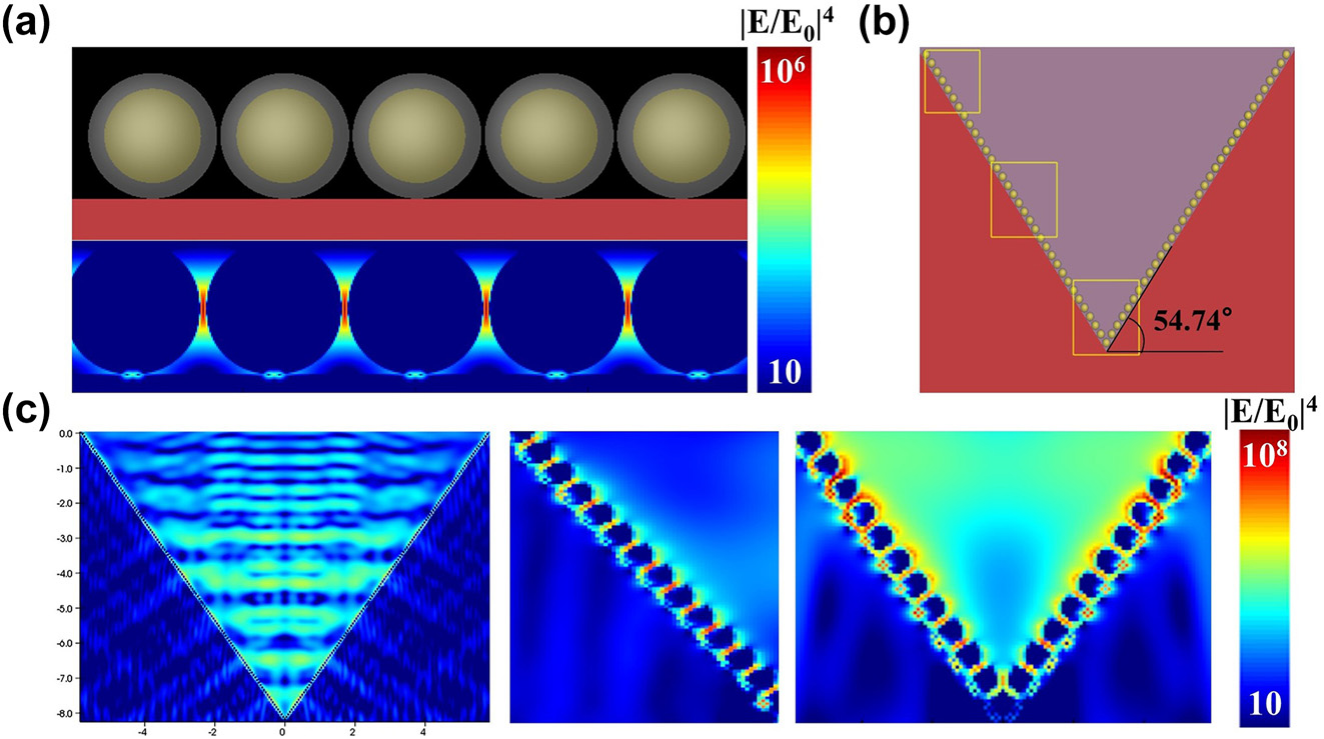
Au@Ag NSs/PPSi composite substrate FDTD simulation model and electromagnetic field distribution. (a) Au@Ag NSs/Si simulation model and electromagnetic field distribution. The intensity of the electromagnetic field |*E*/*E*_0_|^4^ was represented by color scale. (b) Au@Ag NSs/PPSi SERS substrate FDTD simulation model. (c) Electromagnetic field distribution of composite SERS substrate with a height of 8.4 μm.

Au@Ag NSs with a diameter of 85 nm and a spacing of 2 nm placed on a Si plate was simulated for comparison in Figure 11(a), while the electric field properties at the top, middle, and bottom of the PPSi attached Au@Ag NSs (diameter of 85 nm, spacing of 2 nm) with the height of 8.4 μm were simulated and recorded as the Figure 11(b) shows. It can be seen clearly that local electric field enhancement of Au@Ag NSs/Si is located between nanospheres, where EM enhancement |*E*/*E*_0_|^4^ was as high as 10^6^. As shown in Figure 11(c), the incident laser generates effective oscillations within the PPSi, which exhibit a strong localized electric field enhancement within the PPSi valley, presenting as a band-like distribution in the *x* − *z* view. Regarding the enhancement mechanism of the 3D composite SERS substrate, a uniform and locally enhanced electromagnetic field hot spot is generated between Au@Ag NSs. Additionally, multiple oscillations of the incident laser within the PPSi valleys result in optical field amplification, creating a high-density three-dimensional active hot spot. The EM enhancement |*E*/*E*_0_|^4^ reached as high as 10^8^, which was mutually corroborated with the measured results of AEF. The Au@Ag NSs/PPSi composite SERS substrate provided a uniform spatial distribution for the detection of analyte molecules within the specified hotspot areas.

## 4 Conclusions

In this study, a 3D composite SERS substrate based on PPSi combined with Au@Ag NSs was prepared using a simple and rapid method. Through successful SERS detection of the reporting molecules R6G and CV, the 3D composite SERS substrate demonstrated excellent uniformity, high sensitivity, and good strengthening performance. Additionally, due to its excellent biocompatibility, the Au@Ag NSs/PPSi SERS substrate significantly enhanced the Raman signal of *S. aureus*, enabling rapid quantitative detection of pathogenic bacteria. The broad-spectrum SERS enhancement of microorganisms by the composite substrate is currently under investigation. If proven to be broad spectrum for various types of microorganisms, it will provide an effective platform for rapid *in situ* detection of microorganisms. This would have significant implications for the diagnosis and treatment of diseases caused by bacteria, viruses, and other microorganisms and hold promising application prospects in diverse fields.
